# Antimicrobial Susceptibility of *Staphylococcus aureus* Isolated from Recreational Waters and Beach Sand in Eastern Cape Province of South Africa

**DOI:** 10.3390/ijerph14091001

**Published:** 2017-09-01

**Authors:** Olufemi Emmanuel Akanbi, Henry Akum Njom, Justine Fri, Anthony C. Otigbu, Anna M. Clarke

**Affiliations:** Microbial Pathogenicity and Molecular Epidemiology Research Group (MPMERG), Department of Biochemistry and Microbiology, University of Fort Hare, Private Bag X1314, Alice 5700, South Africa; hnjom@ufh.ac.za (H.A.N.); jfri@ufh.ac.za (J.F.); aotigbu@gmail.com (A.C.O.); aclarke@ufh.ac.za (A.M.C.)

**Keywords:** *S. aureus*, antibiotic resistance, beaches, multiple-antibiotic resistance

## Abstract

*Background*: Resistance of *Staphylococcus aureus* to commonly used antibiotics is linked to their ability to acquire and disseminate antimicrobial-resistant determinants in nature, and the marine environment may serve as a reservoir for antibiotic-resistant bacteria. This study determined the antibiotic sensitivity profile of *S. aureus* isolated from selected beach water and intertidal beach sand in the Eastern Cape Province of South Africa. *Methods*: Two hundred and forty-nine beach sand and water samples were obtained from 10 beaches from April 2015 to April 2016. *Staphylococcus aureus* was isolated from the samples using standard microbiological methods and subjected to susceptibility testing to 15 antibiotics. Methicillin-resistant *Staphylococcus aureus* (MRSA) was detected by susceptibility to oxacillin and growth on Brilliance MRSA II agar. Antibiotic resistance genes including *mec*A, *fem*A *rpo*B, *bla*Z, *erm*B, *erm*A, *erm*C, *van*A, *van*B, *tet*K and *tet*M were screened. *Results*: Thirty isolates (12.3%) were positive for *S. aureus* by PCR with over 50% showing phenotypic resistance to methicillin. Resistance of *S. aureus* to antibiotics varied considerably with the highest resistance recorded to ampicillin and penicillin (96.7%), rifampicin and clindamycin (80%), oxacillin (73.3%) and erythromycin (70%). *S. aureus* revealed varying susceptibility to imipenem (96.7%), levofloxacin (86.7%), chloramphenicol (83.3%), cefoxitin (76.7%), ciprofloxacin (66.7%), gentamycin (63.3%), tetracycline and sulfamethoxazole-trimethoprim (56.7%), and vancomycin and doxycycline (50%). All 30 (100%) *S. aureus* isolates showed multiple antibiotic-resistant patterns (resistant to three or more antibiotics). The *mec*A, *fem*A, *rpo*B, *bla*Z, *erm*B and *tet*M genes were detected in 5 (22.7%), 16 (53.3%), 11 (45.8%), 16 (55.2%), 15 (71.4%), and 8 (72.7%) isolates respectively; *Conclusions*: Results from this study indicate that beach water and sand from the Eastern Cape Province of South Africa may be potential reservoirs of antibiotic-resistant *S. aureus* which could be transmitted to exposed humans and animals.

## 1. Introduction

*Staphylococcus aureus* are Gram-positive cocci ranging from 0.5 to 1.5 μm in diameter, which may or may not contain a polysaccharide capsule. They are non-motile, non-spore forming facultative anaerobes that produce catalase and coagulase enzymes [1,2,3]. Yearly, microbial contamination of marine waters is predicted to be responsible for millions of gastrointestinal and acute respiratory infections (ARIs) [4], in addition to several skin infections [5]. Although *S. aureus* is typically a commensal organism, it has been known to be opportunistic. Invasive infections due to wound invasion can lead to numerous diseases, including scalded skin syndrome, abscesses, septicaemia, pneumonia, food poisoning, and toxic shock syndrome [6,7].

*S. aureus* is a potentially harmful human pathogen associated with both nosocomial and community-acquired infections, and it is increasingly becoming resistant to most antibiotics. Previous studies of *S. aureus* in marine environments have linked swimmers to the dissemination of *S. aureus* in marine water [8], via the shedding of the bacterium from their nose, skin, and respiratory tract [9]. On recreational beaches, *S. aureus* has occasionally been found in high abundance in both water and sand, which can be directly associated with bather density and human activities around the beach [9,10,11].

The human skin is directly exposed to infectious agents during swimming [12], and this exposure can lead to the colonization of *S. aureus* with the potential to invade the immune system and cause infections. There is a relationship between seawater exposure and *S. aureus* infection rates which suggests that recreational waters are potential sources of community-acquired *S. aureus* infections [9]. There is also a positive correlation between the concentrations of *S. aureus* and total staphylococci to skin, eye, and ear infections among bathers [13,14,15].

Methicillin-resistant *Staphylococcus aureus* (MRSA) is defined as any strain of *S. aureus* that has acquired resistance to methicillin and other beta lactam antibiotics [16] and it is responsible for several intractable infections in humans [17]. *S. aureus* and MRSA are both shed by swimmers [18,19] and have been reported in beach seawater and sand [18,20,21,22,23,24].

The resistance of *S. aureus* to methicillin is due to the production of penicillin-binding protein 2a (PBP2a), which is encoded by the *mec*A gene located on the mobile gene element (MGE) of the staphylococcal chromosome cassette *mec* (SCC*mec*), which has a low affinity for beta-lactam antibiotics [25,26].

The fact that *S. aureus* is resistant to multiple classes of antimicrobial agents in the hospital environment is a challenge currently facing clinicians when treating *S. aureus* infections [27]. This resistance stems from a history of over 50 years of recurrent adaptation of *S. aureus* to different antibiotics introduced into clinical practice over the years. Abuse of as well as indiscriminate use of antimicrobials are contributing factors to the spread of resistance [27]. Antibiotic-resistance genes are carried on plasmids and transposons, and can be transferred from one staphylococcal species to another and among other Gram-positive bacteria [28].

Antimicrobials act by targeting important bacterial functions such as cell wall synthesis (beta-lactams and glycopeptides), protein synthesis (aminoglycosides, tetracyclines, macrolides, lincosamides, chloramphenicol, mupirocin and fusidic acid), nucleic acid synthesis (quinolones), RNA synthesis (rifampin), and metabolic pathways such as folic acid metabolism (sulphonamides and trimethoprim) [29,30,31]. The overuse of antimicrobials elicits resistance either by the emergence of point mutations or by the acquisition of foreign resistance genes, which leads to alteration of the antimicrobial target and the degradation of the antimicrobial or reduction of the cell’s internal antimicrobial concentration [27,29,30,31].

This study was carried out to determine the antimicrobial resistance pattern of *Staphylococcus aureus* and methicillin-resistant *Staphylococcus aureus* isolated from seawater and sand from selected beaches in the Eastern Cape Province of South Africa. We also determined whether isolates carried any antibiotic-resistance gene markers for methicillin, beta-lactams, tetracycline, vancomycin, erythromycin and rifampicin.

## 2. Materials and Methods 

### 2.1. Study Site

**Figure 1:**
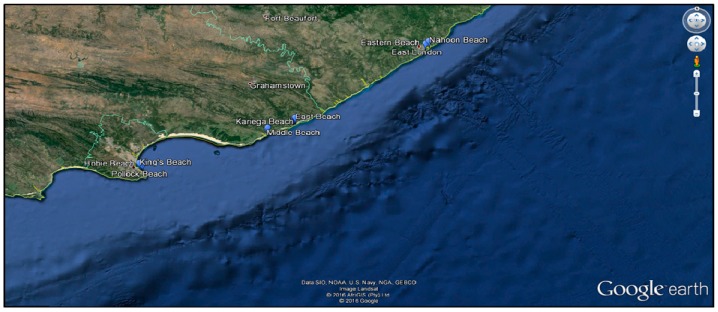
Aerial view of sampling sites [32].

Sea water and sand samples were obtained from ten beaches in four major cities in the Eastern Cape Province; Nahoon beach (32°59′20.09′′ S 27°57′1.30′′ E), Eastern beach (33°0′32.00′′ S 27°55′31.02′′ E), East beach (33°36′6.07′′ S 26°54′4.94′′ E), West beach (33°36′18.80′′ S 26°53′56.53′′ E), Kelly’s beach (33°36′37.20′′ S 26°53′25.86′′ E), Kariega beach (33°41′1.05′′ S 26°40′59.28′′ E), Middle beach (33°41′21.16′′ S 26°40′36.09′′ E), King’s beach (33°58′16.92′′ S 25°38′49.87′′ E), Hobie beach (33°58′49.75′′ S 25°39′35.18′′ E), and Pollock beach (33°59′6.59′′ S 25°40′21.92′′ E) (Figure 1).

### 2.2. Sample Collection

A total of 245 (178 marine water, 67 marine sand) samples were collected monthly from 10 selected beaches in the Eastern Cape Province of South Africa between April 2015 and April 2016. Water samples were collected in 2 L sterile containers against an incoming wave. Beach sand was also collected in sterile 100 mL containers. Samples were transported at 4 °C and processed within 24 h.

#### Isolation and Molecular Confirmation of *S. aureus*

Sand samples were vigorously hand shaken in Phosphate Buffered Saline (PBS), where a ratio of 2 g of sand to 80 mL of PBS was used [20,33]. Both sand and water samples were enriched in tryptone soy broth and incubated at 37 °C for 24 h, followed by sub-culturing on mannitol salt agar (MSA), and further incubated at 37 °C for 24 h. Presumptive *S. aureus*, identified by the fermentation of mannitol (yellow colonies) were purified on nutrient agar. Presumptive isolates were stored in 25% glycerol at −80 °C.

Polymerase chain reaction (PCR) was used for confirmation of *S. aureus* as previously described [20]. DNA was extracted using the boiling method where 2 mL of overnight pure Nutrient broth cultures were transferred to sterile eppendorf tubes and centrifuged at 13,000 rpm for 3 min. The supernatant was discarded and cells re-suspended in 200 μL sterile distilled water. The cell solution was then heated at 100 °C in an Accu dri-block (Lasec, SA) for 10 min, followed by centrifugation at 13,000 rpm for 2 min to pellet the cells [34]. The supernatants were transferred to clean, sterile tubes and used directly as templates for PCR assay or stored at −20 °C for subsequent use.

A method previously described by Maes [35] was used for identification of *S. aureus*, based on the detection of a specie-specific nuc-gene. *S. aureus* ATCC 25923 was used as a positive control. Each 25 μL PCR reaction mix constituted 12.5 μL of 2X PCR master mix, 0.5 μL each of both reverse and forward primers (Table 1), 6.5 μL nuclease-free water and 5 μL of template DNA. PCR was conducted in a T1000 Touch Thermal Cycler (Bio-Rad, Hercules, CA, USA). Cycling conditions are shown in Table 1. The PCR products were separated by agarose gel electrophoresis in 1% agarose, stained with ethidium bromide. A 100 bp DNA ladder was included in each run.

### 2.3. Antimicrobial Susceptibility Testing

Isolates confirmed by PCR as *S. aureus* were subjected to antimicrobial susceptibility testing to 15 antibiotics. Profiling was performed by the Kirby-Bauer disk diffusion method on Mueller-Hinton agar according to Clinical and Laboratory Standards Institute guidelines [36,37]. An inoculum for each isolate was prepared by emulsifying colonies from an overnight pure culture in sterile normal saline (0.85%) in test tubes with the turbidity adjusted to 0.5 McFarland standard (0.5 mL of 1% *w*/*v* BaCl_2_ and 99.5 mL of 1% *v*/*v* H_2_SO_4_), equivalent to 1.0 × 10^8^ cfu/mL. The bacterial suspension was uniformly streaked on Mueller Hinton agar plates using sterile swabs and left for 3 min prior to introduction of the antibiotics. Antibiotics commonly used for treatment of *S. aureus* infections were selected for this assay, namely penicillin, ampicillin, gentamycin, erythromycin, levofloxacin, ciprofloxacin, tetracycline, doxycycline, vancomycin, cefoxitin, imipenem, sulfamethoxazole-trimethoprim, clindamycin, rifampicin and chloramphenicol. Plates were incubated at 35 °C for 24 h, and the diameters of zone of inhibition were measured and results interpreted according to Clinical Laboratory Standards institute [37].

### 2.4. Detection of MRSA

#### 2.4.1. Phenotypic

All isolates confirmed to be *S. aureus* by PCR were subjected to antibiotic susceptibility testing to oxacillin (5 μg) [37] by disc diffusion test as well as growth on Brilliance MRSA II agar [38], to determine phenotypic resistance to methicillin. Inoculated plates were incubated at 37 °C for 24 h [38]. All isolates that tested positive on Brilliance MRSA II agar (blue to violet colonies) or resistant by oxacillin disc were considered to be presumptive MRSA.

#### 2.4.2. Molecular Confirmation of MRSA

Presumptive isolates from Brilliance MRSA II agar, as well as isolates that were phenotypically resistant to oxacillin, were further confirmed by PCR detection of the *mec*A gene (responsible for methicillin resistance) using specific primers (Table 1) as earlier described [20,39]. The *fem*A gene, a factor essential for methicillin resistance, was also evaluated [40] by PCR using specific primers (Table 1). A 25 uL reaction was set up consisting of 12.5 μL master mix, 0.5 μL forward primer, 0.5 μL reverse primer, 6.5 μL nuclease-free water and 5 μL of DNA. PCR was conducted using a T1000 Touch Thermal Cycler (Bio-Rad, Johannesburg, SA, USA). The cycling conditions used for confirmation of the *mec*A and *fem*A gene are shown in Table 1**.** The amplicons were separated using 1.5% agarose stained with ethidium bromide and visualized under a transilluminator (UVITEC Alliance 4.7, Bio-Active., Ltd., Bangkok, Thailand).

### 2.5. PCR Detection of Antibiotic Resistance Genes

Based on the phenotypic antibiotic resistance profiles, (29/30, 24/30, 22/30, 21/30, 17/30, 15/30) isolates showing resistance to β-lactam, rifampicin, methicillin, erythromycin, tetracycline, vancomycin respectively, were investigated for the presence of associated antibiotic-resistance genes (ARGs). These were *bla*Z, *rpo*B, *mec*A *erm*B, *erm*A, *erm*C, *tet*K, *tet*M, *van*A and *van*B genes respectively. The reactions were performed as singleplex PCRs in a total volume of 25 μL consisting of 12.5 μL 2X PCR master mix, 0.5 μL each of the forward and reverse primer, 6.5 μL nuclease-free water and 5 μL of template DNA performed in a T1000 Touch Thermal Cycler (Bio-Rad, Johannesburg, SA, USA). The amplicons were separated on 1.5% agarose stained with ethidium bromide, visualized and photographed using a transilluminator (UVITEC Alliance 4.7, Bio-Active., Ltd., Bangkok, Thailand). Table 1 shows the primer sequences used, and cycling conditions for PCR detection of *S. aureus* and antibiotic resistance gene markers.

## 3. Results

### 3.1. Molecular Identification of Staphylococcus aureus in Recreational Beach Water and Sand Samples

A total of 245 samples were screened; beach water (*n* = 178) and sand samples (*n* = 67) of which 143 isolates (one isolate from each sample) were presumptive by culture on MSA. A 12.3% (30/245) of the isolates were confirmed by PCR as *S. aureus*, with 12.4% (22/178) of isolates from seawater, and 11.9% (8/67) from marine sand. Of the 22 confirmed *S. aureus* isolates from seawater, 6 isolates each were from Middle beach and Eastern beach, 5 isolates from Nahoon beach, 2 each from Kariega beach and East beach and 1 isolate from West beach. Of the 8 confirmed *S. aureus* isolates from sand, 4 isolates were from Middle beach and 2 each were from East beach and Kariega beach respectively.

### 3.2. Antimicrobial Susceptibility Test (AST)

**Figure 2:**
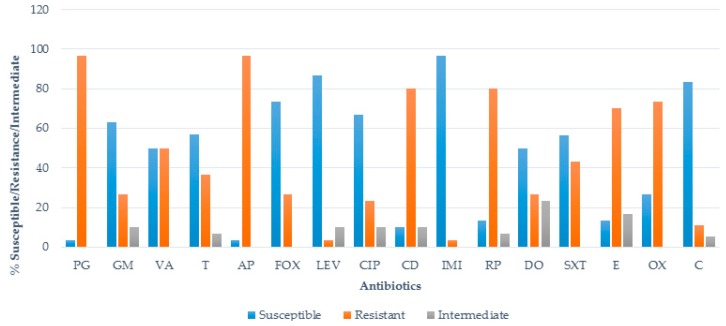
The percentage of antimicrobial resistance profiles of *S. aureus* isolates. PG = penicillin, GM = gentamicin, VA = vancomycin, T = tetracycline, AP = ampicillin, FOX = cefoxitin, LEV = levofloxacin, CIP = ciprofloxacin, CD = clindamycin, IMI = imipenem, RP = rifampicin, DO = doxycycline, SXT = sulfamethoxazole-trimethoprim, E = erythromycin, OX = oxacillin, C = chloramphenicol.

Antibiotic susceptibility of 30 *S. aureus* isolates revealed varying degrees of susceptibility patterns against the antimicrobial agents. Generally, cefoxitin 76.7% (23/30), chloramphenicol 83.3% (25/30), levofloxacin 86.7% (26/30), and imipenem 96.7% (29/30) were the most effective antibiotics to *S. aureus.* A low, ≥50% susceptibility was recorded to vancomycin and doxycycline (50%; 15/30), tetracycline and sulfamethoxazole-trimethoprim (56.7%; 17/30), gentamycin (63.3%; 19/30), and ciprofloxacin (66.7%; 20/30). A higher resistance to erythromycin (70%; 21/30) and clindamycin and rifampicin (80%, 24/30) was identified, with resistance to penicillin G and ampicillin the highest (each recording 96.7%; 29/30). The percentage of antimicrobial resistance of *S. aureus* isolates are shown in Figure 2.

### 3.3. Phenotypic Detection of MRSA

A methicillin-resistant *S. aureus* isolate was defined as resistant by any of the two methods tested. Fifteen (50%) isolates showed phenotypic resistance to methicillin after culturing on selective media (Brilliance MRSA II agar) while 73.3% (22/30) of the isolates showed phenotypic resistance to oxacillin (Figure 2), which could be used as a proxy for determining methicillin resistance [46]. All those that were positive on Brilliance MRSA II agar were also positive for the oxacillin disc diffusion test.

### 3.4. Multiple Antibiotic Resistance (MAR)/MAR Phenotypes of S. aureus

All isolates tested were multi-drug resistant, (100%; 30/30) (resistant to three or more antimicrobials), with 3 isolates resistant to 12 of the 15 antibiotics tested. Resistance to 8 antibiotics was the most common, shown by 5 (16.7%) isolates, followed by resistance to 4 and 5 antibiotics recorded by 4 (13.3%) isolates each. Twenty-three different MAR patterns were observed from the 30 isolates. The most common of these were PG-GM-VA-T-AP-FOX-CIP-CD-RP-DO-SXT-E-OX, PG-VA-T-AP-FOX-CD-RP-DO-SXT-E-OX and PG-VA-AP-FOX-CD-RP-SXT-E-OX, observed in 3 (10%), 2 (6.7%), and 2 (6.7%) isolates, respectively.

### 3.5. Prevalence of Antibiotic Resistance Genes

**Figure 3:**
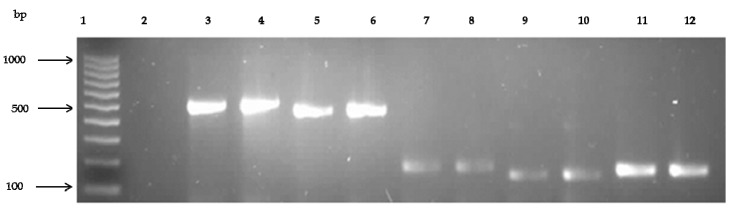
Representative gel showing PCR amplified products of antibiotic resistance genes of *mec*A, *rpo*B, *bla*Z, *erm*B and *tet*M separated on 1.5% agarose. Lane 1: 100 bp DNA ladder (Fermentas Life Sciences, Vilnius, Lithuania), Lane 2: negative control, Lane 3, 4: *mec*A (499 bp) positive isolates, Lane 5, 6: *rpo*B (460 bp) positive isolates, Lane 7, 8: *bla*Z (173 bp) positive isolates, Lane 9, 10: *erm*B (142 bp) positive isolates and Lane 11, 12: *tet*M (142 bp) positive isolates.

Generally, a total of five of 10 ARGs tested were detected in one or more resistant isolates, with higher frequencies recorded in isolates recovered from seawater. Of the ten ARGs tested (*bla*Z, *mec*A, *rpo*B, *erm*B, *erm*A, *erm*C, *tet*K, *tet*M, *van*A and * van*B), the *blaZ* gene, coding for resistance to beta-lactam antibiotics (penicillin & ampicillin), was detected in 16 (55.2%, *n* = 29) of the isolates, the *mec*A gene, coding for methicillin resistance was detected in 5 (22.7%, *n* = 22), the *rpo*B gene, coding for rifampicin resistance, was detected in 11 (45.8%, *n* = 24), the *erm*B gene, coding for erythromycin resistance, in 15 (71.4%, *n* = 21) and the *tet*M gene, coding for tetracycline resistance, was detected in 8 (72.7%, *n* = 11) of the isolates. However, other ARGs such as *erm*A, *erm*C, *tet*K, *van*A and *van*B investigated were absent in the isolates. Table 2 shows the various ARGs detected in beach sand and water while Figure 3 shows a representative gel of the PCR amplified products for these genes.

**Figure 4:**
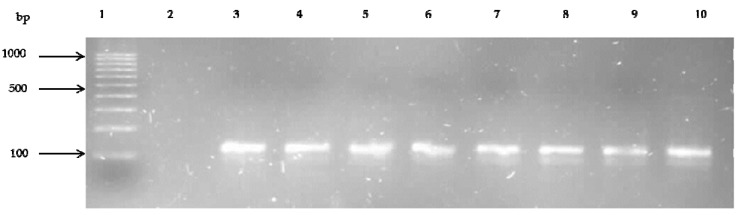
Representative gel showing PCR amplified products of *fem*A gene separated on 1.5% agarose. Lane 1: 100 bp DNA ladder (Fermentas Life Sciences, Vilnius, Lithuania), Lane 2: negative control, Lane 3–10: *fem*A (132 bp) positive isolate.

The * fem*A gene, a factor also responsible for methicillin resistance [40], was identified in 53.3% (16/30) of the isolates. Figure 4 shows the gel electrophoresis of PCR amplified products for the *fem*A gene.

## 4. Discussion

Humans and animals have been reported as sources of antibiotic-resistant organisms in water environments and can transfer antibiotic resistance genes to other pathogens and naturally occurring water microbes through transposons, plasmids and integrons [47,48]. Bacteria isolated from beach sand, seawater and sediments have recorded resistance to various antimicrobials [48,49,50,51,52].

The occurrence of *S. aureus* and MRSA is on the rise, resulting in increased incidences of hospital-acquired and community-acquired infections worldwide, posing a major public health concern [53,54,55]. Moreover, microbial ecosystems can also be potentially altered by the presence of varying antibiotics of industrial origin, circulating in water environs [47]. *S. aureus* is one of the most successful and adaptable human pathogens due to its proficiency in acquiring antibiotic-resistant mechanisms and pathogenic determinants, leading to its emergence in both nosocomial and community settings [54]. Nosocomial colonisation of *S. aureus* and MRSA can go undetected, and signs of infection may only appear months after a patient is exposed to the infection. Infected patients may then serve as reservoirs for further transmission, especially as most of these strains carry SCC*mec* types coding for resistance to methicillin and other beta lactams [56].

To the best of our knowledge, this is the first study which has used a mixture of phenotypic and genotypic approaches simultaneously to determine the occurrence and antibiotic resistance profiles of *S. aureus* strains from beach water and sand in the study area. In this study, *S. aureus* was isolated from beach water and sand samples. Other studies have also reported this organism in marine water and/or sand [18,20,21,22,24,57,58,59], however, the frequency (12.2%) of isolation was lower in our study than observed in other studies [20,21,24]. This study only analyzed a single isolate for every sample, which could account for the lower detection frequency.

Of the isolates evaluated in this study, individual resistances of *S. aureus* to penicillin G and ampicillin was high (96.7%; 29/30). High resistance to these β-lactam antibiotics was not surprising, as ampicillin is one of the most commonly used antibiotics for treatment of infections in humans and animals [60], with penicillin developing resistance to *S. aureus* since the 1960s [61]. In addition, ampicillin-resistant isolates may cross-select for resistance to other beta-lactams [62]. Resistance to ampicillin may therefore indicate resistance of the isolates to other β-lactam antibiotics. This was observed in our study, as resistance to both ampicillin and penicillin occurred in equal proportion. Resistances observed to erythromycin, chloramphenicol, sulfamethoxazole-trimethoprim, and tetracycline were similar to that previously reported [21].

Given the relatively small number of isolates evaluated, a 50% vancomycin resistance was of concern, as this antibiotic is historically regarded as the antibiotic of final resort and the highest quality level antimicrobial for the treatment of genuine MRSA diseases [30]. The first case of a fully vancomycin-resistant *S. aureus* was described in Michigan, USA, in a renal dialysis patient [63]. The utilization of growth promoters such as tylosin, macrolide and avoparcin has been related to the occurrence of erythromycin and vancomycin resistance in *S. aureus* [64] in the environment, which might have then leached to marine waters. Based on the phenotypic identification of MRSA, 50% and 73.3% of the isolates were potentially MRSA by both methods. The poor specificity of the phenotypic methods in this study was not surprising, as higher specificity and sensitivity of these phenotypic methods have mostly been recorded in clinical isolates [38].

In this study, all *S. aureus* isolates were multidrug resistant. This point is worth noting, as it potentially could lead to failure in treatment therapy, prolonged illnesses, increased expenses for health care, and in serious cases, risk of death if humans are infected with such strains [65]. The transmission of resistance (R-factor), a plasmid-mediated genetic determinant, may be credited with the development of MAR among these isolates [62]. Studies have shown an upward pattern in the incidences of *S. aureus* isolates with multiple antibiotic resistance [66,67,68,69]. It has also been reported that *S. aureus* isolates with multiple antibiotic resistance attributes have a negative impact on the treatment of staphylococcal infections, especially in elderly, children, and immune-compromised individuals [70].

Generally, a total of five out of 10 ARGs tested were detected, with a higher frequency of detection in beach water compared to sand isolates. The higher frequency of detection in seawater could be because water is exposed to a greater variety of potential contaminants than sand. These may include runoffs from pharmaceutical, hospital, and industrial waste as well as farmlands [71,72]. Sources may also include antibiotic-resistant bacteria from poorly treated or untreated sewage, as final effluents of waste water treatment plants that may leach into seawater [52].

The *bla*Z gene is responsible for the production of β- lactamase enzyme, which confers resistance to β- lactam antibiotics such as penicillin and ampicillin [73]. This gene was only detected in small proportions compared to its phenotypic detection. Molecular confirmation identified the *mec*A gene only in five (22.7%) of the MRSA isolates detected by at least one of the phenotypic methods. The presence of this gene encodes a penicillin-binding protein 2a (PBP2a), responsible for methicillin resistance in staphylococci, with this protein, rendering a reduced affinity for β-lactam antibiotics [74]. Various studies have reported the occurrence of methicillin-resistant *S. aureus* from water sources, animal-derived food and humans [55,75,76]. MRSA has also been previously reported from marine waters [21,24] and waste water treatment plants [77]. Oxacillin has been proposed as a proxy antibiotic for testing susceptibility not only to methicillin and to all β-lactams [46], which could explain why all oxacillin-resistant isolates were not carrying the *mec*A gene. Phenotypic resistance observed to oxacillin in this study was probably achieved through other mechanisms [78], which may include alteration of the penicillin binding proteins, which brings about hyper-production of methicillinase or beta-lactamase [37,79,80].

The mode of resistance of rifampin is inhibition of the process of RNA polymerase [81]. Mutations on the gene encoding the β-subunit of RNA polymerase (*rpo*B gene) account for rifampin resistance (Rif^r^) [82,83,84]. In our study, this gene was detected in 45.8% (11/24) of the rifampicin-resistant *S. aureus* isolates. Erythromycin resistance in staphylococci is mainly facilitated by the *erm* genes, coding for erythromycin resistant methylase [85], with *erm*A and *erm*C reported as the most frequently detected *erm* gene associated with staphylococci in human infections [86]. Results from this study however, detected *erm*B as the only gene coding for erythromycin resistance. The high incidence (72.7%) of *tet*M in our study is similar to that (74.2%) earlier reported [87]. Another study has also reported the presence of both *tet*M and *tet*K gene from *S. aureus* isolates from public beaches [21].

The *fem*A gene was detected in 53.3% (16/30) of the confirmed *S. aureus* isolates. This gene is a chromosomally encoded factor in *Staphylococcus aureus*, which is crucial for the expression of advanced methicillin resistance, encoding proteins which influence the level of methicillin resistance [88]. Finding *fem*A gene in all *mec*A positive isolates is evidence that these isolates had a functional methicillin resistance. The detection of *fem*A together with *mec*A by PCR has long been considered a reliable indicator in the identification of MRSA [89].

## 5. Conclusions

This study is the first to report the occurrence of antibiotic resistant *S. aureus* on recreational beaches in the Eastern Cape Province, South Africa. Our results show that public beaches in the study area may be potential reservoirs for transmission of antibiotic resistant *S. aureus* to beach goers, particularly those with skin lesions. Results from this study are unlikely to be unique to the Eastern Cape or South Africa and further studies are needed to determine the distribution and level of antibiotic-resistant *S. aureus* in other public beaches.

## Figures and Tables

**Table 1:** Oligonucleotide primers and cycling conditions used in the molecular confirmation of *S. aureus* and antibiotic-resistance genes.

Primer	Sequence (5′-3′)	Product Size (bp)	Cycling Conditions	Reference
nuc-F	GCGATTGATGGTGGATACGGT	279	Initial denaturation at 94 °C for 5 min, followed by 40 cycles of 94 °C for 45 s, 58 °C for 45 s and 72 °C for 90 s. Final extension at 72 °C for 10 min	[35]
nuc-R	AGCCAAGCCTTGACGAACTAAAGC			
mecA-F	TCCAGGAATGCAGAAAGACCAAAGC	499	Initial denaturation at 94 °C for 3 min, followed by 40 cycles of 94 °C for 30 s, 59 °C for 30 s and 72 °C for 1 min. Final extension at 72 °C for 8 min.	[41]
mecA-R	GACACGATAGCCATCTTCATGTTGG			
ermA-F	TATCTTATCGTTGAGAAGGGATT	139	Initial denaturation at 94 °C for 5 min, followed by 40 cycles of 94 °C for 40 s, 48 °C for 40 s and 72 °C for 90 s. Final extension at 72 °C for 8 min.	[42]
ermA-R	CTACACTTGGCTTAGGATGAAA			
ermB-F	CTATCTGATTGTTGAAGAAGGATT	142	Initial denaturation at 94 °C for 5 min, followed by 40 cycles of 94 °C for 40 s, 47 °C for 40 s and 72 °C for 90 s. Final extension at 72 °C for 8 min.	[42]
ermB-R	GTTTACTCTTGGTTTAGGATGAAA			
ermC-F	CTTGTTGATCACGATAATTTCC	190	Initial denaturation at 94 °C for 5 min, followed by 40 cycles of 94 °C for 40 s, 49 °C for 40 s and 72 °C for 90 s. Final extension at 72 °C for 8 min.	[42]
ermC-R	ATCTTTTAGCAAACCCGTATTC			
blaZ-F	ACTTCAACACCTGCTGCTTTC	173	Initial denaturation at 94 °C for 3 min, followed by 35 cycles of 94 °C for 30 s, 49 °C for 30 s and 72 °C for 1 min. Final extension at 72 °C for 8 min.	[42]
blaZ-R	TGACCACTTTTATCAGCAACC			
rpoB1-F	ACCGTCGTTTACGTTCTGTA	460	Initial denaturation at 94 °C for 5 min, followed by 40 cycles of 94 °C for 40 s, 45.5 °C for 40 s and 72 °C for 90 s. Final extension at 72 °C for 8 min.	[43]
rpoB2-R	TCAGTGATAGCATGTGTATC			
tetM-F	AGTGGAGCGATTACAGAA	158	Initial denaturation at 94 °C for 3 min, followed by 40 cycles of 94 °C for 30 s, 45 °C for 30 s and 72 °C for 1 min. Final extension at 72 °C for 8 min.	[44]
tetM-R	CATATGTCCTGGCGTGTCTA			
tetK-F	GTAGCGACAATAGGTAATAGT	360	Initial denaturation at 94 °C for 3 min, followed by 40 cycles of 94 °C for 30 s, 47 °C for 30 s and 72 °C for 1 min. Final extension at 72 °C for 8 min.	[44]
tetK-R	GTAGTGACAATAAACCTCCTA			
vanA	GCGCGGTCCACTTGTAGATA	314	Initial denaturation at 94 °C for 3 min, followed by 35 cycles of 94 °C for 1 min, 56.5 °C for 1 min and 72 °C for 1 min. Final extension at 72 °C for 10 min.	[45]
vanA	TGAGCAACCCCCAAACAGTA			
vanB	AGACATTCCGGTCGAGGAAC	220	Initial denaturation at 94 °C for 3 min, followed by 35 cycles of 94 °C for 1 min, 56.5 °C for 1 min and 72 °C for 1 min. Final extension at 72 °C for 10 min.	[45]
vanB	GCTGTCAATTAGTGCGGGAA			
femA-F	AAAAAAGCACATAACAAGCG	132	Initial denaturation at 94 °C for 5 min, followed by 40 cycles of 94 °C for 40 s, 45.5 °C for 40 s and 72 °C for 90 s. Final extension at 72 °C for 8 min.	[40]
femA-R	GATAAAGAAGAAACCAGCAG			

**Table 2:** Antibiotic resistance genes detected in *S. aureus* isolates from beach sand and seawater.

No. Resistant by Disc Diffusion	Associated ARG Tested	ARG Detected		
		Sand (%)	Water (%)	Total (%)
Ampicillin & Penicillin (*n* = 29)	*bla*Z	4 (25%)	12 (75%)	16 (55.2%)
Methicillin (*n* = 22)	*mec*A	1 (20%)	4 (80%)	5 (22.7%)
Rifampicin (*n* = 24)	*rpo*B	2 (18.2%)	9 (81.8%)	11 (45.8%)
Erythromycin (*n* = 21)	*erm*B	3 (20%)	12 (80%)	15 (71.4%)
Tetracycline (*n* = 11)	*tet*M	1 (12.5%)	7 (87.5%)	8 (72.7%)
